# Spatial association of an AKR1C3-positive epithelial state with ACTA2-positive stromal cells and SPP1-positive macrophages in gastric cancer

**DOI:** 10.3389/fonc.2026.1965632

**Published:** 2026-10-28

**Authors:** Jingjing Yang, Yixuan Dai, Jianpeng Sheng, Rong Xu

**Affiliations:** 1 Zhangjiagang Hospital Affiliated to Soochow University/The First People's Hospital of Zhangjiagang City, Zhangjiagang, Jiangsu, China; 2 Multi-Omics Platform, Capital Medical University Innovation Center, Beijing, China

**Keywords:** AKR1C3, gastric cancer, single-cell RNA-sequencing, spatial transcriptomics, tumor microenvironment

## Abstract

**Introduction:**

The spatial organization of epithelial states relative to stromal and myeloid cells in gastric cancer remains incompletely understood.

**Methods:**

We integrated single-cell RNA-sequencing data from 36 normal and tumor tissue samples (GSE183904) with Visium spatial transcriptomics (GSE251950). Cell-state signatures were evaluated in TCGA-STAD (n = 375), and multiplex immunohistochemistry was performed on specimens from three patients.

**Results:**

Epi4_AKR1C3 and FB3_ACTA2 showed nominal enrichment in tumor tissues, whereas Mac2_SPP1 showed no significant compositional difference between normal and tumor tissues. Higher signature scores for these three states were nominally associated with shorter overall survival (log-rank P = 0.044, 0.019, and 0.048, respectively), although none remained significant after multiple-testing correction. Spatial deconvolution identified regions of co-enrichment, and multiplex immunohistochemistry provided proof-of-concept evidence of close spatial proximity among the corresponding marker-defined populations.

**Discussion:**

These findings identify a candidate AKR1C3-associated epithelial–stromal–myeloid spatial configuration in gastric cancer. Further mechanistic studies are needed to validate its biological and clinical significance.

## 1 Introduction

Gastric cancer (GC) remains both prevalent and deadly today. The Global Cancer Observatory estimated > 968,000 new cases and approximately 660,000 deaths in 2022 (1). Outcomes tend to be poorer for patients who present with advanced or metastatic GC, even with aggressive combinations of treatment, including the use of immune checkpoint blockade in the first-line setting (2–4). The challenge of GC patient treatment is in part due to the cancer’s heterogeneity. The Lauren classification describes intestinal, diffuse, and mixed histological types (5), and The Cancer Genome Atlas (TCGA) describes Epstein–Barr virus (EBV)-positive, microsatellite-unstable (MSI) tumors, genomically stable (GS) tumors, and chromosomally unstable (CIN) tumors (6). These groups vary not just in terms of their molecular drivers, but also in the makeup and structure of the tumor microenvironment (TME), with effects on prognosis and treatment responses (6–8).

The gastric TME is the result of cooperation between epithelial cells, cancer-associated fibroblasts (CAFs), and tumor-associated macrophages (TAMs) (6–11). There are multiple functional states of CAFs, but we find the ACTA2-high, TGF-β1-responsive myofibroblast-like state of special interest. This state is involved in matrix production and immune suppression and resists immune checkpoint blockade in gastrointestinal cancers (10–12). Similar to CAFs, there are also multiple macrophage states. SPP1-positive TAMs are involved in immune-restrictive niches and extracellular matrix (ECM) remodeling in GC (8, 13). Studies of peritoneal metastasis have also identified a complement-dependent macrophage-CAF axis as an immunomodulatory pathway that can modulate immune checkpoint blockade responsiveness (11). Hence, there is an accumulating body of evidence that stromal-myeloid interactions are important in GC, whereas a connection to particular epithelial states is less clear.

One question that has largely been overlooked is whether a distinct epithelial transcriptional state is spatially associated with a nearby fibroblast–macrophage community. In this context, AKR1C3 may serve as a representative marker of a broader epithelial gene program. This aldo-keto reductase has been studied for chemoresistance, intracrine androgen synthesis, and the metabolism of prostaglandins and steroids. It has also been linked to GC invasion and metastasis (14, 15). Arachidonic-acid-derived mediators can also influence Th2-type inflammation and immune regulation in the TME (14). An AKR1C3-positive epithelial program may thus create an immunomodulatory and lipid-abundant tissue environment, although it could just as easily be a marker of such an environment. It has not yet been shown whether these epithelial cells migrate close to myofibroblast-like CAFs and SPP1-high TAMs.

Spatial transcriptomics can help us resolve this question concerning location. Data from the Visium studies reveal an immunosuppressive fibroblast-to-macrophage network in GC (9). However, there is a key limitation to the platform. Each capture spot includes RNA from many different cells. Co-enrichment at the spot level is insufficient to demonstrate that two cells are in fact adjacent to each other. Ligand-receptor algorithms cannot prove that the sending cell is the proposed sender cell either (16, 17). So, it is necessary to subject evidence from single-cell state definitions to regional analysis and finally histological examination at the cellular level.

Here, data from GSE251950’s Visium were merged with GSE183904’s single-cell RNA-sequencing (scRNA-seq) data to analyze the spatial relationship of epithelial cells, fibroblasts, and macrophages. NicheNet (16) ranked the epithelial-to-stromal and epithelial-to-myeloid categories, and The Cancer Genome Atlas Stomach Adenocarcinoma (TCGA-STAD) offered a preliminary assessment of survival-related associations. Independently, multiplex immunohistochemistry provided proof-of-concept cellular-resolution evidence of marker localization.

## 2 Materials and methods

### 2.1 Datasets and data access

GSE183904 was the single-cell reference dataset (7). The dataset contains 36 tissues profiled on the 10x Genomics Chromium platform: 10 normal samples and 26 tumor samples from gastric sites. Spatial studies employed GSE251950, for which processed feature-barcode matrices and histological images are available from 10 Visium sections belonging to nine GC cases (9). For the exploratory survival studies only, we downloaded harmonized RNA-seq and clinical data for TCGA-STAD from the Genomic Data Commons (GDC) Data Portal (https://portal.gdc.cancer.gov/) (6).

### 2.2 scRNA-seq preprocessing and integration

A cell was retained when 200–6,000 genes were detected and mitochondrial unique molecular identifiers (UMIs) accounted for < 20% of its library; genes detected in < three cells were removed. DoubletFinder v.2.0.3 was applied with an expected doublet rate of 0.08 (18). We log-normalized counts with a scale factor of 10,000 and selected 2,000 highly variable genes by variance-stabilizing transformation. Canonical correlation analysis in Seurat was then used to integrate the samples (19). The first 20 principal components were carried forward to Uniform Manifold Approximation and Projection (UMAP) and the shared-nearest-neighbor graph (n.neighbors = 30, min.dist = 0.3). Leiden clusters were obtained with FindClusters at resolution 0.8. Marker testing used FindAllMarkers and the Wilcoxon rank-sum test, with log2 fold change ≥ 0.25 and a minimum detection rate of 0.25. Lineages were assigned from canonical markers reported in GC single-cell studies (7, 8). We subsequently re-clustered each major lineage at a resolution suited to that compartment. The resulting subclusters were defined as transcriptionally distinct cell states and were named using representative marker genes. For example, Epi4_AKR1C3 denotes epithelial state 4, in which AKR1C3 is a representative marker used for cluster annotation. The same nomenclature was applied to other cell state labels. Monocle 3 was used for the epithelial trajectory with the default learn_graph and order_cells settings (20). InferCNV v.1.8.1 provided an estimate of broad copy-number patterns (21). We used immune cells from the same sample as the diploid reference.

### 2.3 Survival analysis

For TCGA-STAD, the 20 most differentially expressed genes in a subpopulation defined its signature. We calculated Gene Set Variation Analysis (GSVA) scores from bulk RNA-seq data (22). A total of 29 cell-state signatures, comprising nine epithelial, four fibroblast, four macrophage, six B-cell, and six T-cell states, were evaluated. Kaplan–Meier curves were generated by median dichotomization and compared using two-sided log-rank tests. Continuous GSVA scores were further evaluated using multivariable Cox regression adjusted for age, sex, tumor purity, and American Joint Committee on Cancer (AJCC) stage, with hazard ratios (HRs), 95% confidence intervals (CIs), *P*-values and FDR-adjusted *q* reported. Benjamini–Hochberg correction was applied across the 29 cell-state survival tests, with FDR-adjusted *q* values considered the primary measure of statistical evidence and nominal *P* values retained for exploratory interpretation.

### 2.4 Gene ontology enrichment

Gene Ontology biological process analysis was performed using clusterProfiler v.4.6.0 (23). For each subpopulation, the input comprised its 20 leading genes drawn from a marker pool with adjusted *P* < 0.05. Hypergeometric testing was used for enrichment. We retained terms with Benjamini–Hochberg (BH)-adjusted *P* < 0.05 and a gene ratio of at least 0.05, after which clusterProfiler::simplify was used to collapse redundant terms. The dot-plot color scale represents -log10 of the raw hypergeometric *P*-values. Inclusion in the plot, by contrast, was determined from the BH-adjusted value.

### 2.5 Spatial transcriptomic analysis

Processed matrices and tissue images for GSE251950 were read into Seurat, and the Visium data were normalized with SCTransform. Robust Cell Type Decomposition (RCTD) v.2.1.0 was run in “full” mode using the single-cell data as its reference (24). Within a section, we called a spot jointly enriched only when Epi4_AKR1C3, FB3_ACTA2, and Mac2_SPP1 were each above their own 50th-percentile estimate. Regional proximity between each pair was summarized by a 10-nearest-neighbor score. Spatial significance was assessed separately within each Visium section using 1,000 within-section label permutations. For each permutation, the spatial coordinates of tissue spots were held fixed, whereas cell-state labels were randomly reassigned among spots within the same section, thereby preserving the section-specific number of spots assigned to each state while disrupting their observed spatial arrangement. No labels were exchanged across sections. The tri-cellular 10-nearest-neighbor (KNN) score was recalculated after each permutation to generate the null distribution, and the observed score was compared with this distribution. The empirical P value was calculated as (1 + number of permuted scores ≥ observed score)/(1 + 1,000). All epithelial–fibroblast–macrophage combinations were then evaluated using the same tri-cellular KNN scoring framework for descriptive comparison of their spatial-association scores.

### 2.6 Ligand-receptor inference

Using NicheNet (16) (NicheNet v.2.1.5), receptor target genes were those differentially expressed from other states of the same lineage (log2 fold change > 0.5; adjusted *P* < 0.05). They were scored against the relevant target set using the human ligand-target matrix v.2. The output is a ranking built from expression and prior interaction knowledge.

### 2.7 Multiplex immunohistochemistry

Tissue sections were made at 4 μm. Staining used the Opal Polaris 7-color Manual IHC Kit (Akoya Biosciences, Marlborough, MA, USA; NEL861001KT). The slides underwent 1 h at 65 °C before dewaxing in xylene and passage through a graded alcohol series. They were then fixed for 20 min in 10% neutral buffered formalin (G2161, Solarbio, China). For antigen retrieval, microwave power was set to 100% for 1 min and 20% for the next 15 min. Slides were allowed to cool and were then blocked for 10 min. Each staining cycle comprised primary antibody, Opal Polymer horseradish peroxidase (HRP) Mouse + Rabbit (Ms + Rb) and the assigned Opal reagent, with Tris-buffered saline with Tween 20 (TBST) washes between steps. Pan-CK, AKR1C3, ACTA2, SPP1, and CD68 occupied separate cycles; the last target used Opal tyramide signal amplification–digoxigenin (TSA-DIG) and Opal Polaris 780. Nuclei were counterstained with 4′,6-diamidino-2-phenylindole (DAPI) (C1002, Beyotime) in Mowiol (81381, Sigma-Aldrich). We acquired images on a Vectra Polaris Quantitative Imaging System. Three independent GC specimens were analyzed, with three tumor regions of interest (ROIs) per patient. The three ROIs obtained from each patient were considered nested spatial observations. Accordingly, the mIHC analysis was interpreted as a proof-of-concept cellular-resolution assessment. Pan-CK^+^/AKR1C3^+^ epithelial cells, ACTA2^+^ stromal cells, and SPP1^+^/CD68^+^ macrophages were quantified. Pairwise cell-to-cell and nearest-neighbor distances were calculated within each ROI and compared with permutation-derived null distributions. Permutation-derived P values were used to evaluate deviation from spatial randomness within individual ROIs. Ratios < 1 indicated shorter-than-random spatial proximity. A representative tumor field is shown, both merged and by single channel, with a 100 μm scale bar. GC specimens from patients were obtained from the Zhangjiagang First People’s Hospital, Affiliated Zhangjiagang Hospital of Soochow University. This study was reviewed and approved by the Medical Ethics Committee of Zhangjiagang First People’s Hospital (approval No. ZJGYYLL-LW-2026-09-010). Written informed consent was obtained from all participants.

### 2.8 Statistical analysis

For cell-state compositional comparisons, two-sided Wilcoxon rank-sum tests were performed at the sample level, followed by Benjamini–Hochberg correction for multiple testing within each lineage. Both nominal and false discovery rate (FDR)-adjusted *P*-values were reported. Computations were run in R 4.3.2 with survival 3.5-5, survminer 0.4.9, and GSVA 1.50.0. *P* < 0.05 was considered the nominal cutoff, whereas FDR-adjusted *q* < 0.05 was considered statistically significant for analyses involving multiple testing.

## 3 Results

### 3.1 Integrated single-cell and spatial profiling maps the GC cellular landscape

To build a complete cellular framework for identifying cell states associated with tumors and their possible spatial interactions, we began by analyzing the cellular composition and tissue organization of the TME of stomach cancer at both the single-cell and spatial resolution levels. The integrated scRNA-seq dataset contained 150,963 cells after quality control. These came from all 36 GSE183904 samples, including 10 normal samples and 26 gastric tumor samples (7). Eight major lineages were identified on the UMAP (**Figures 1A–C**). We assigned them as T cells (CD3G), epithelial cells (EPCAM), B cells (MZB1), fibroblasts (COL1A1), macrophages (CD68), endothelial cells (PECAM1), dendritic cells (CD1C), and mast cells (GATA2). When these lineages were considered in aggregate, their proportions differed between normal (N) and tumor (T) samples (**Figure 1D**). To account for inter-sample variability, we further assessed major lineage proportions at the individual sample level, with the corresponding distributions and FDR-adjusted results shown in **Figure 1E** and **Supplementary Figure S2A**.

**Figure 1:**
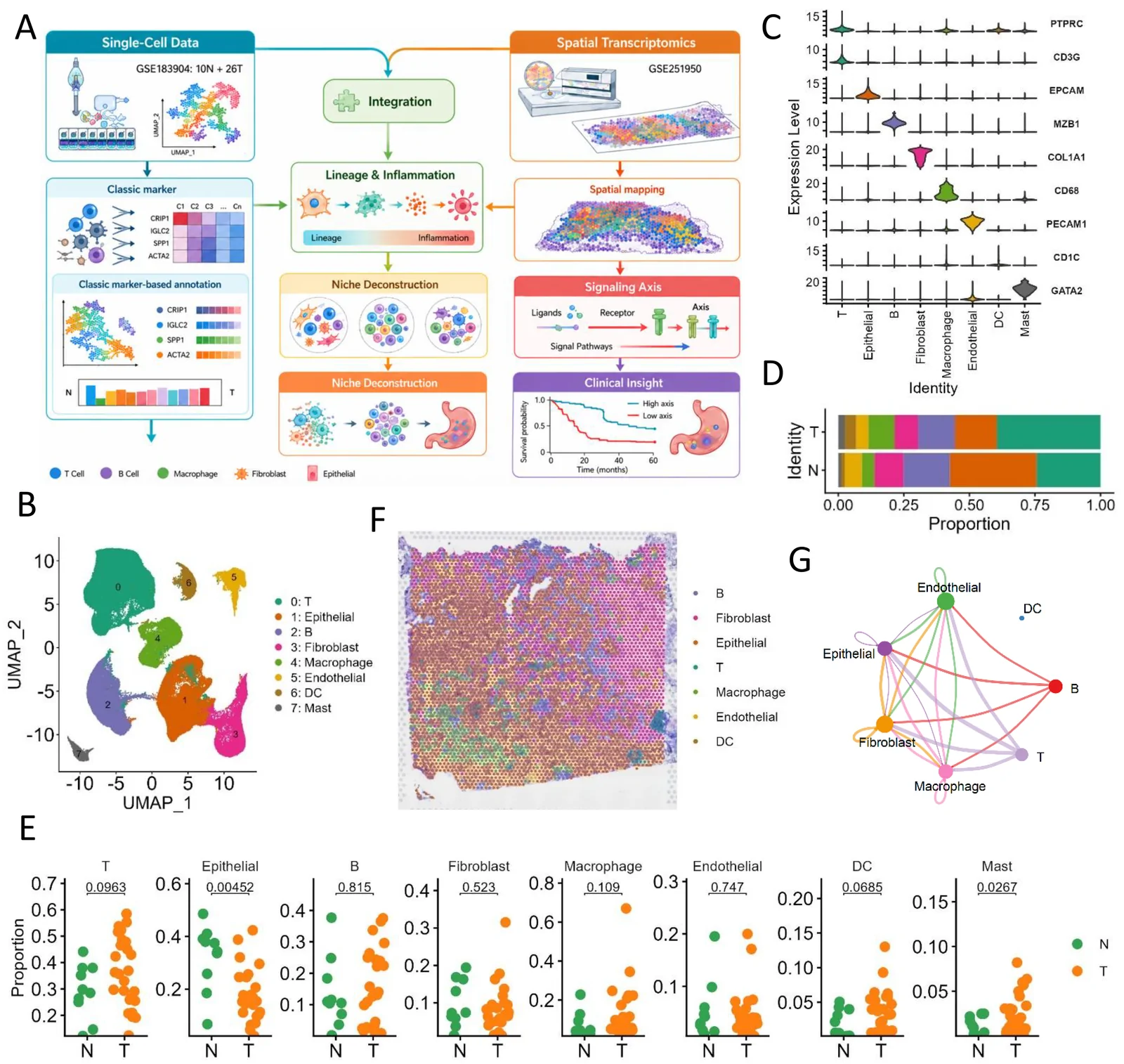
Single-cell and spatial transcriptomic atlas of the GC TME. **(A)** Study workflow showing scRNA-seq (GSE183904; 10 normal and 26 tumor tissue samples) and Visium spatial transcriptomics (GSE251950). **(B)** UMAP of 150,963 high-quality cells, colored by major lineage. **(C)** Violin plots of canonical lineage markers. **(D)** Stacked bar chart of aggregate major-lineage composition in normal (N) versus tumor (T) samples. **(E)** Per-sample subpopulation proportions; nominal *P*-values are from two-sided Wilcoxon rank-sum tests. **(F)** RCTD-estimated abundance maps on a representative Visium tumor section. **(G)** CellChat-inferred ligand-receptor co-expression networks across major lineages; edge thickness scales with the number of supporting pairs.

Projection onto GSE251950 with RCTD added a spatial view to the single-cell atlas. The major lineages varied markedly across each section, indicating substantial spatial heterogeneity, with epithelial, fibroblast, macrophage, and other lineage signals partially overlapping in regions (**Figure 1F**). Since each Visium spot encompasses transcripts from many cells, this phenomenon is understood as regional co-enrichment. CellChat analysis at the major lineage level yielded a complementary expression-based overview of possible inter-lineage communication (**Figure 1G**), representing lineage-specific gene expression alongside previously obtained data with respect to ligand-receptor interactions. The spatial structure of major populations was outlined by RCTD, while CellChat disclosed the potential biological compatibility.

### 3.2 Epi4_AKR1C3 marks a tumor-associated epithelial state with distinct metabolic features

Because epithelial cells are responsible for malignant processes associated with GC and are likely to initiate tumor-associated interactions with stromal and immune cells, we first resolved epithelial heterogeneity at greater resolution. Nine epithelial states, Epi1-Epi9, were recovered on re-clustering and named using representative marker genes (**Figures 2A, B**). Among the nine subpopulations, the inferCNV distribution was not uniform (**Figure 2C**). Relative to the immune-cell reference, Epi9_CDH11 and Epi7_CXCL11 had the highest scores, while Epi3_PGA3 had the lowest. The others lay between them, above Epi3_PGA3 but below the strongly aneuploid states. This gradient of the inferred burden of copy number variation (CNV) suggests that there exists considerable genomic heterogeneity in epithelial tissue, from a state close to being diploid, as shown by Epi3_PGA3, to more extensively damaged states represented by Epi7_CXCL11 and Epi9_CDH11. The various intermediate CNV profiles of these cellular populations indicate that gastric epithelial cells exist along a continuum of genomic alterations rather than as a distinct set of normal versus highly aneuploid populations. Most importantly, this gradient provides a genomic framework for identifying epithelial states even if it does not indicate whether a malignant state is reached.

**Figure 2:**
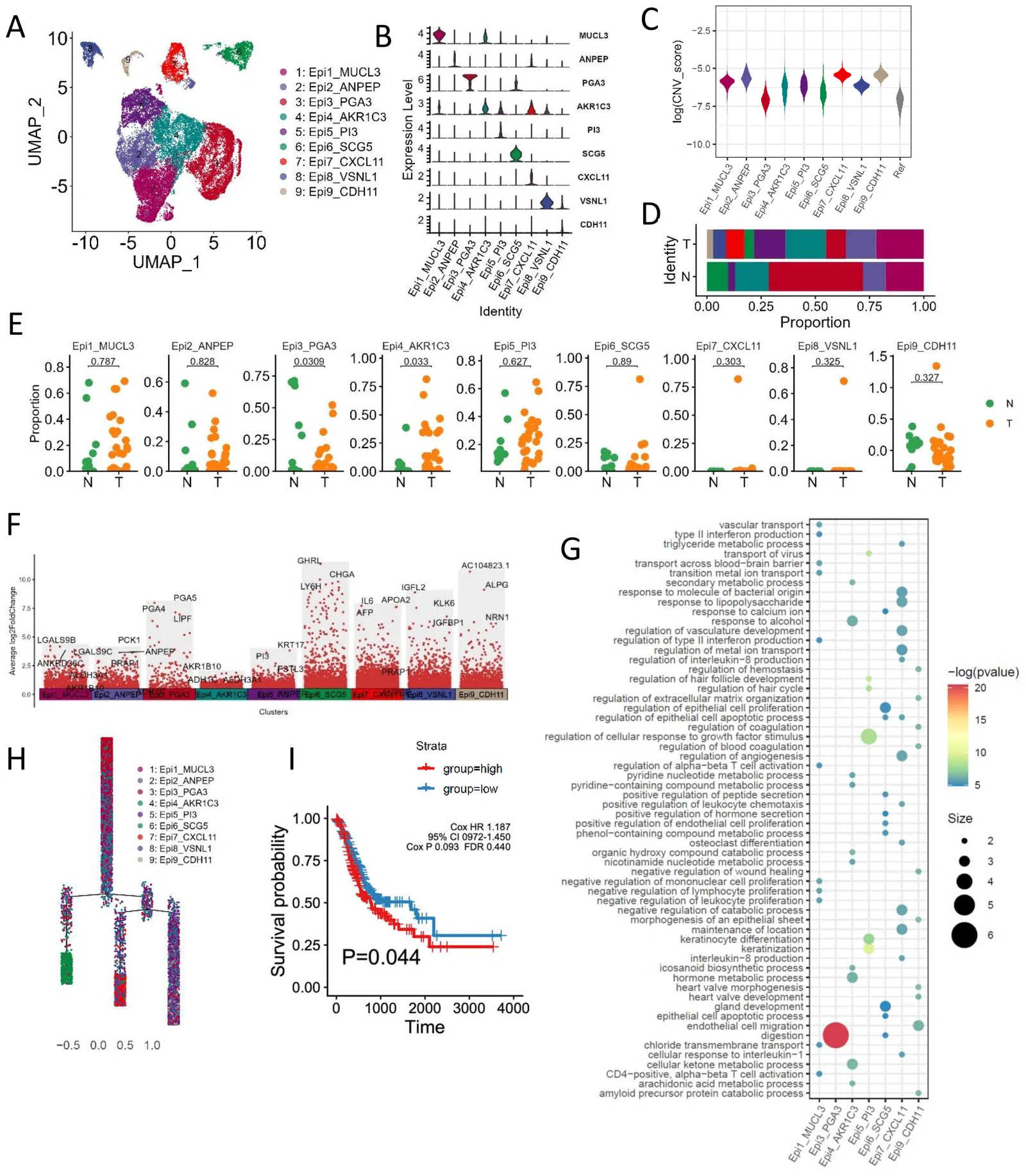
Epi4_AKR1C3 is an AKR1C3-positive epithelial state with nominal tumor enrichment and an exploratory survival association. **(A)** UMAP of 24,709 epithelial cells. **(B)** Violin plots of marker genes. **(C)** Per-state inferCNV scores with immune cells as the diploid reference. **(D)** Stacked bar chart of aggregate epithelial-subpopulation composition in N versus T samples. **(E)** Per-sample subpopulation proportions; nominal *P*-values are from two-sided Wilcoxon rank-sum tests. **(F)** Top marker-gene expression. **(G)** GO biological-process terms passing BH-adjusted *P* < 0.05; dot color represents −log10 of the raw hypergeometric *P*-value. **(H)** Monocle 3 pseudotime trajectory. **(I)** Kaplan-Meier overall survival in TCGA-STAD stratified at the median Epi4_AKR1C3 GSVA score.

At the sample level, only two epithelial states differed at the nominal compositional threshold (**Figures 2D, E**). Neither epithelial-state comparison remained statistically significant after within-lineage FDR correction (**Supplementary Figure S2A**). At the nominal level, Epi4_AKR1C3 was more abundant in tumors (P = 0.033), whereas Epi3_PGA3 favored normal samples (P = 0.031). The other seven comparisons were negative. Their marker genes and Gene Ontology (GO) terms further separated the states (**Figures 2F, G**). Digestion-related functions were retained in Epi3_PGA3. Epi4_AKR1C3 carried nicotinamide- and arachidonic-acid-related transcripts, Epi7_CXCL11 had inflammatory and angiogenic programs, and Epi9_CDH11 was associated with ECM remodeling and epithelial morphogenesis. These findings indicate that epithelial heterogeneity extends beyond differences in inferred CNV burden to encompass distinct functional programs, motivating further evaluation of whether specific epithelial states, particularly the tumor-enriched Epi4_AKR1C3 population, may contribute to remodeling of the surrounding TME. Furthermore, Epi4_AKR1C3 occupied an intermediate position on the Monocle 3 trajectory, between Epi3_PGA3 and Epi7_CXCL11/Epi9_CDH11 (**Figure 2H**). Patients with higher Epi4_AKR1C3 signature scores exhibited shorter overall survival by Kaplan–Meier analysis (log-rank *P* = 0.044), while the association showed an adverse trend but did not reach statistical significance in multivariable Cox regression (HR = 1.187, 95% CI = 0.972–1.450, P = 0.093; BH-FDR q = 0.440; **Figure 2I**). The survival association was therefore considered exploratory. Taken together, Epi4_AKR1C3 was prioritized for downstream spatial analysis based on its nominal tumor enrichment, distinct metabolic transcriptional program, intermediate position along the epithelial trajectory, and exploratory survival association.

### 3.3 FB3_ACTA2 represents a tumor-enriched, myofibroblast-like stromal state

Fibroblasts have become a major focus of TME research because they influence matrix remodeling, paracrine communication, immune regulation, and treatment response. We therefore examined fibroblast heterogeneity in greater detail to identify stromal states enriched in gastric tumors and potentially relevant to disease progression. The 13,779 fibroblasts separated into four states: FB1_PDGFRA (matrix fibroblast), FB2_CXCL12 (inflammatory fibroblast), FB3_ACTA2 (myofibroblast), and FB4_CD74 (antigen-presenting fibroblast) (**Figures 3A, B**). None of the four fibroblast states remained statistically significant after within-lineage FDR correction (**Supplementary Figure S2A**). At the nominal level, FB3_ACTA2 was more abundant in tumor samples (P = 0.047, **Figures 3C, D**), whereas the other three fibroblast states showed no nominal differences. Vasculature development and cell adhesion appeared among the FB3_ACTA2 GO terms, fitting a stromal-remodeling program (**Figures 3E, F**). Together, these features are consistent with a myofibroblast stromal state associated with active remodeling and structural reorganization of the gastric TME. We next assessed the FB3_ACTA2 signature in TCGA-STAD. Although this association did not remain statistically significant after multiple-testing correction (FDR = 0.440; **Figure 3G**), higher FB3_ACTA2 signature scores remained nominally associated with shorter overall survival after multivariable adjustment (HR = 1.241, 95% CI = 1.051–1.445, P = 0.021), suggesting that the FB3_ACTA2 state may mark a stromal context and was therefore interpreted as an exploratory clinical association.

**Figure 3:**
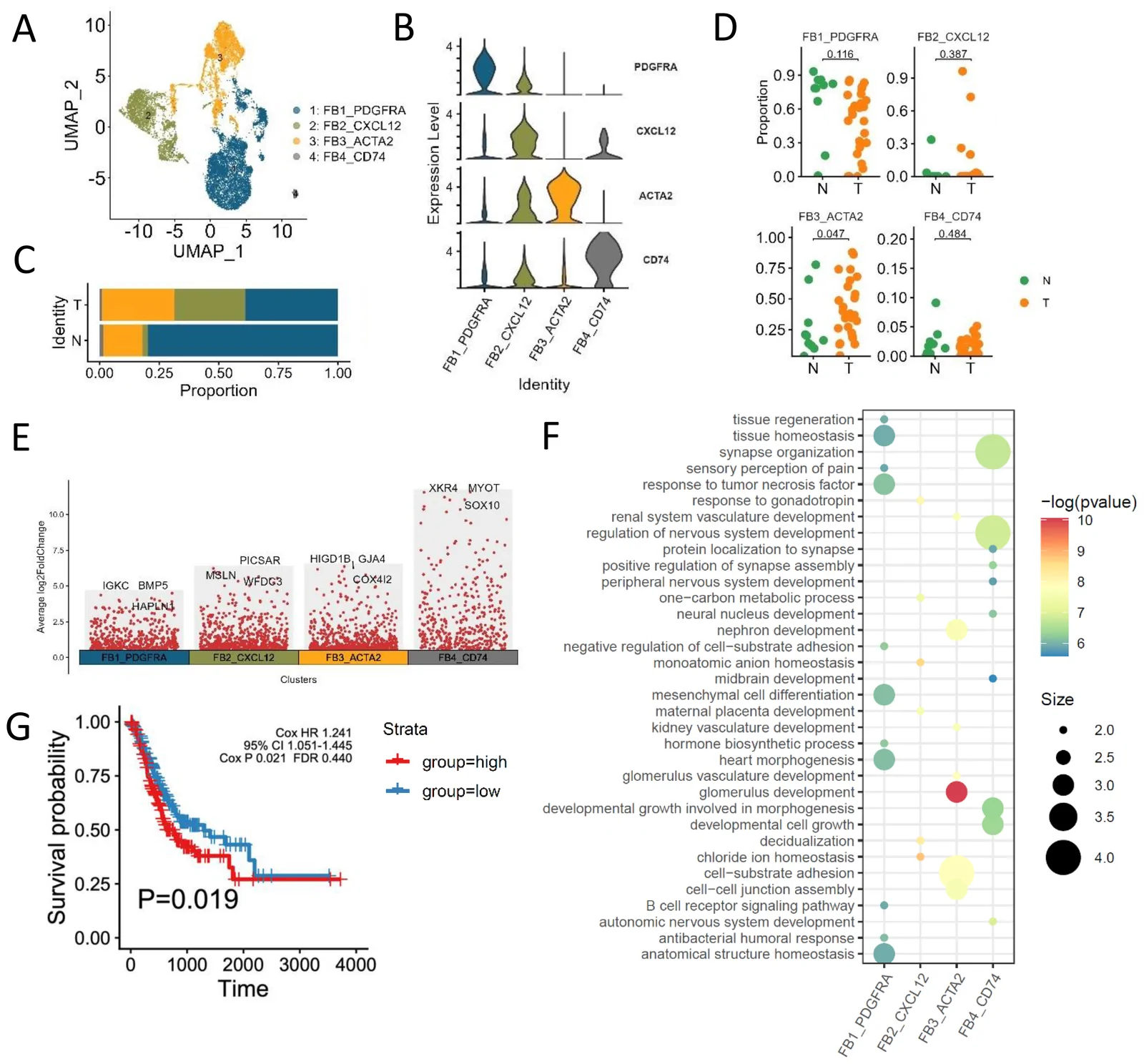
FB3_ACTA2 is an ACTA2-high fibroblast state with nominal tumor enrichment and an exploratory survival association. **(A)** UMAP of 13,779 fibroblasts. **(B)** Violin plots of characteristic genes. **(C)** Stacked bar chart of aggregate fibroblast-subpopulation composition in N versus T samples. **(D)** Per-sample subpopulation proportions; nominal *P*-values are from two-sided Wilcoxon rank-sum tests. **(E)** Top marker gene expression. **(F)** GO biological process terms passing BH-adjusted *P* < 0.05; dot color represents −log10 of the raw hypergeometric *P*-value. **(G)** Kaplan-Meier overall survival in TCGA-STAD stratified at the median FB3_ACTA2 GSVA score.

### 3.4 Mac2_SPP1 identifies an SPP1-high macrophage state with phagocytic and tissue-remodeling features

Given the central role of macrophages in shaping inflammation, immune suppression, tissue remodeling, and tumor progression, we next examined their heterogeneity in the GC microenvironment, focusing on functionally distinct myeloid states. Among 12,580 macrophages, re-clustering recovered four states: Mac1_MRC1, Mac2_SPP1, Mac3_S100A8, and Mac4_C1QA (**Figures 4A, B**). Their abundance did not differ nominally between normal (N) and tumor (T) samples (**Figures 4C, D**; **Supplementary Figure S2A**). Thus, macrophage involvement in the TME appeared not to be driven by overt changes in subpopulation abundance, raising the possibility that functional state rather than abundance may be more informative.

**Figure 4:**
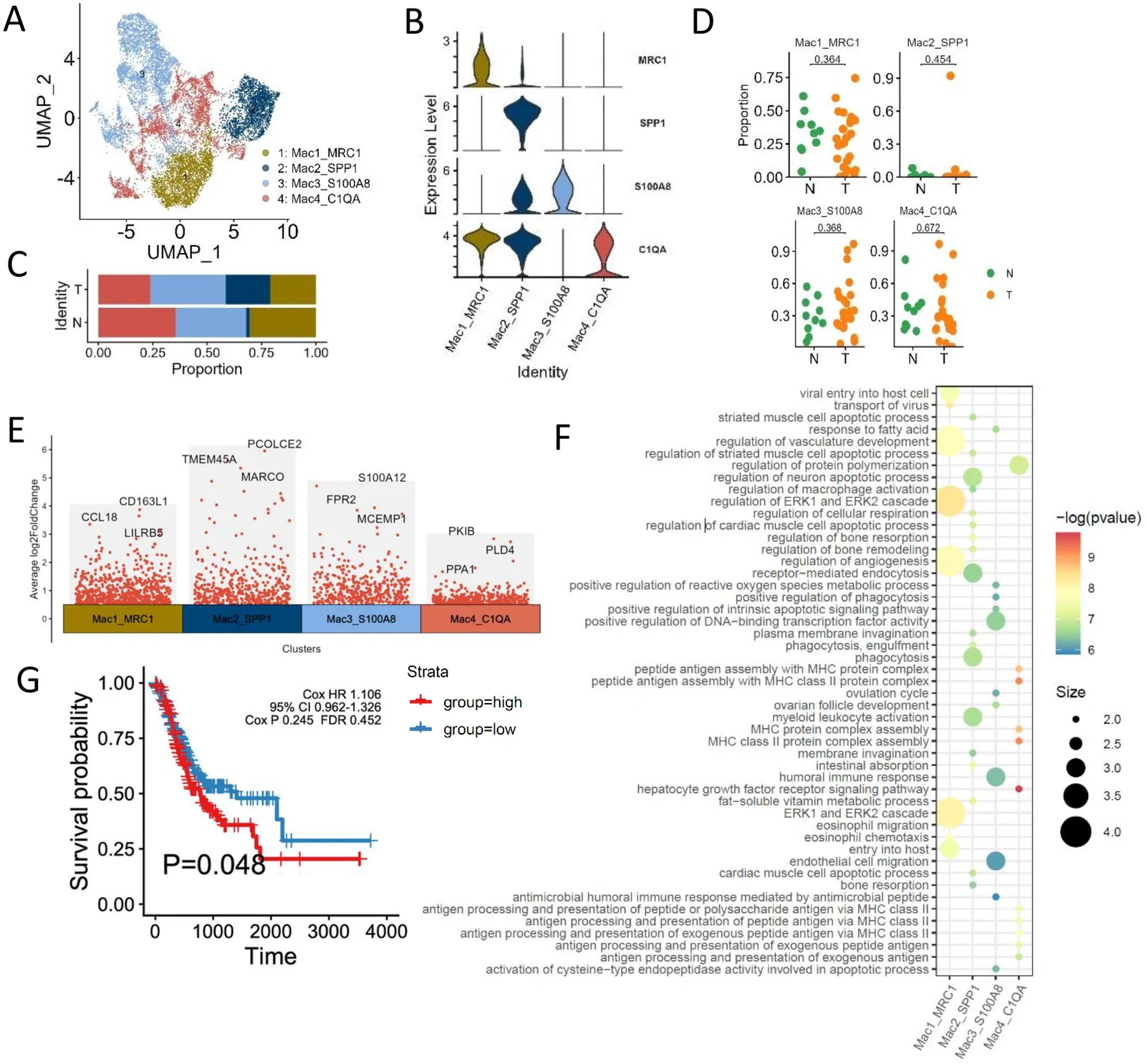
Mac2_SPP1 defines an SPP1-high macrophage state. **(A)** UMAP of 12,580 macrophages. **(B)** Violin plots of marker genes. **(C)** Stacked bar chart of aggregate macrophage-subpopulation composition in N versus T samples. **(D)** Per-sample subpopulation proportions with nominal *P*-values from two-sided Wilcoxon rank-sum tests. **(E)** Top marker-gene expression. **(F)** GO biological process terms passing BH-adjusted *P* < 0.05; dot color represents −log10 of the raw hypergeometric *P*-value. **(G)** Kaplan-Meier overall survival in TCGA-STAD stratified at the median Mac2_SPP1 GSVA score.

Given the absence of marked differences in macrophage-state abundance between normal and tumor tissues, we next analyzed whether these populations were distinguished instead by their transcriptional and functional programs. Marker expression and GO profiles differed across the four states (**Figures 4E, F**). Mac2_SPP1 expressed SPP1 and carried terms related to receptor-mediated endocytosis, positive regulation of phagocytosis, phagocytic engulfment, membrane invagination, and myeloid-cell activation. This pattern resembles phagocytic, matrix-remodeling SPP1-positive macrophages previously described in GC (8, 13). Moreover, higher Mac2_SPP1 signature scores showed an exploratory survival trend (log-rank *P* = 0.048), although the association was not statistically significant in multivariable Cox regression (HR = 1.106, 95% CI = 0.962–1.326, P = 0.245; BH-FDR q = 0.452; **Figure 4G**), and Mac2_SPP1 abundance itself did not differ between normal and tumor samples. This contrast may reflect the macrophage program rather than macrophage number. Taken together, these results define Mac2_SPP1 as an SPP1-high macrophage state with phagocytic and tissue-remodeling features. Its possible contribution to tumor progression may reflect this transcriptional program rather than preferential accumulation in tumor tissue.

### 3.5 Adaptive immune profiling reveals concurrent enrichment of IL7R^+^ memory-like and FOXP3^+^ regulatory T-cell states

Since adaptive immune cells strongly influence antitumor responses, we also examined B- and T-cell heterogeneity to determine whether lymphocyte programs differed in GC. The adaptive immune analysis resolved six B-cell states and six T-cell states (**Supplementary Figures S1A–D**). However, no B-cell state was nominally enriched in either normal (N) or tumor (T) samples, and the overall composition was similar (**Supplementary Figures S1E, G**, **S2A**). Their marker and GO profiles were dominated by immunoglobulin-related and other immune-response programs (**Supplementary Figures S1I, K**).

Changes involving the T-cell compartment were modest (**Supplementary Figures S1F, H**). None of the six T-cell states remained statistically significant after within-lineage FDR correction (**Supplementary Figure S2A**). At the nominal level, tumor samples contained higher proportions of T1_IL7R memory-like cells (P = 0.034; FDR q = 0.103) and T4_FOXP3 regulatory cells (P = 0.012; FDR q = 0.072), whereas the other four T-cell states showed no nominal differences. T-cell activation, cytokine response, and regulatory functions were all represented in the GO results (**Supplementary Figures S1J, L**). Thus, memory/activation-associated and immunoregulatory programs were present at the same time. No B-cell or T-cell signature was nominally associated with overall survival in TCGA-STAD. These findings indicate adaptive immune changes within the broader GC microenvironment, although none of the B- or T-cell states displayed a clear prognostic association in our datasets. We therefore focused subsequent analyses on the epithelial, stromal, and myeloid states that showed more pronounced tumor-associated and clinical patterns.

### 3.6 Spatial mapping identifies regional co-enrichment of Epi4_AKR1C3, FB3_ACTA2, and Mac2_SPP1

Based on the lineage-specific characterization described above, Epi4_AKR1C3, FB3_ACTA2, and Mac2_SPP1 were prioritized as candidate epithelial, stromal, and myeloid states for subsequent spatial analysis. The TCGA-STAD survival analysis was treated as exploratory supporting evidence. Across the 29 cell-state signatures evaluated, the nominal survival associations of these three states did not remain statistically significant after FDR correction (**Supplementary Table S1**). We next focused on whether these populations were spatially co-enriched within gastric tumor tissue. The spatial maps showed considerable intratumoral heterogeneity. Epi4_AKR1C3, FB3_ACTA2, and Mac2_SPP1 were unevenly distributed, although some tumor regions contained relatively high estimates of all three states (**Figure 5A**). We defined co-enriched spots as those in which each state exceeded its section-specific 50th percentile. Such spots occurred within shared tumor regions, indicating regional overlap among the candidate epithelial, fibroblast, and macrophage populations. We then examined pairwise proximity using 10-nearest-neighbor analysis. The scores varied across cell-state pairs and were modest for Epi4_AKR1C3, FB3_ACTA2, and Mac2_SPP1 (**Figure 5B**), whereas the strongest associations were generally observed between epithelial states, showing that these states can occur in the same tumor regions. Spatial randomization analysis showed that the observed tri-cellular KNN spatial-association score for Epi4_AKR1C3, FB3_ACTA2, and Mac2_SPP1 exceeded the randomized null distribution (empirical *P* = 0.001; **Supplementary Figure S3A**), indicating that their regional spatial association was unlikely to be explained by the within-section permutation null model alone. Comparison with alternative epithelial–fibroblast–macrophage combinations further showed that the Epi4_AKR1C3–FB3_ACTA2–Mac2_SPP1 triad ranked among the higher-scoring combinations, highlighting it as a prominent spatial configuration among the combinations evaluated (**Supplementary Figure S3B**).

**Figure 5:**
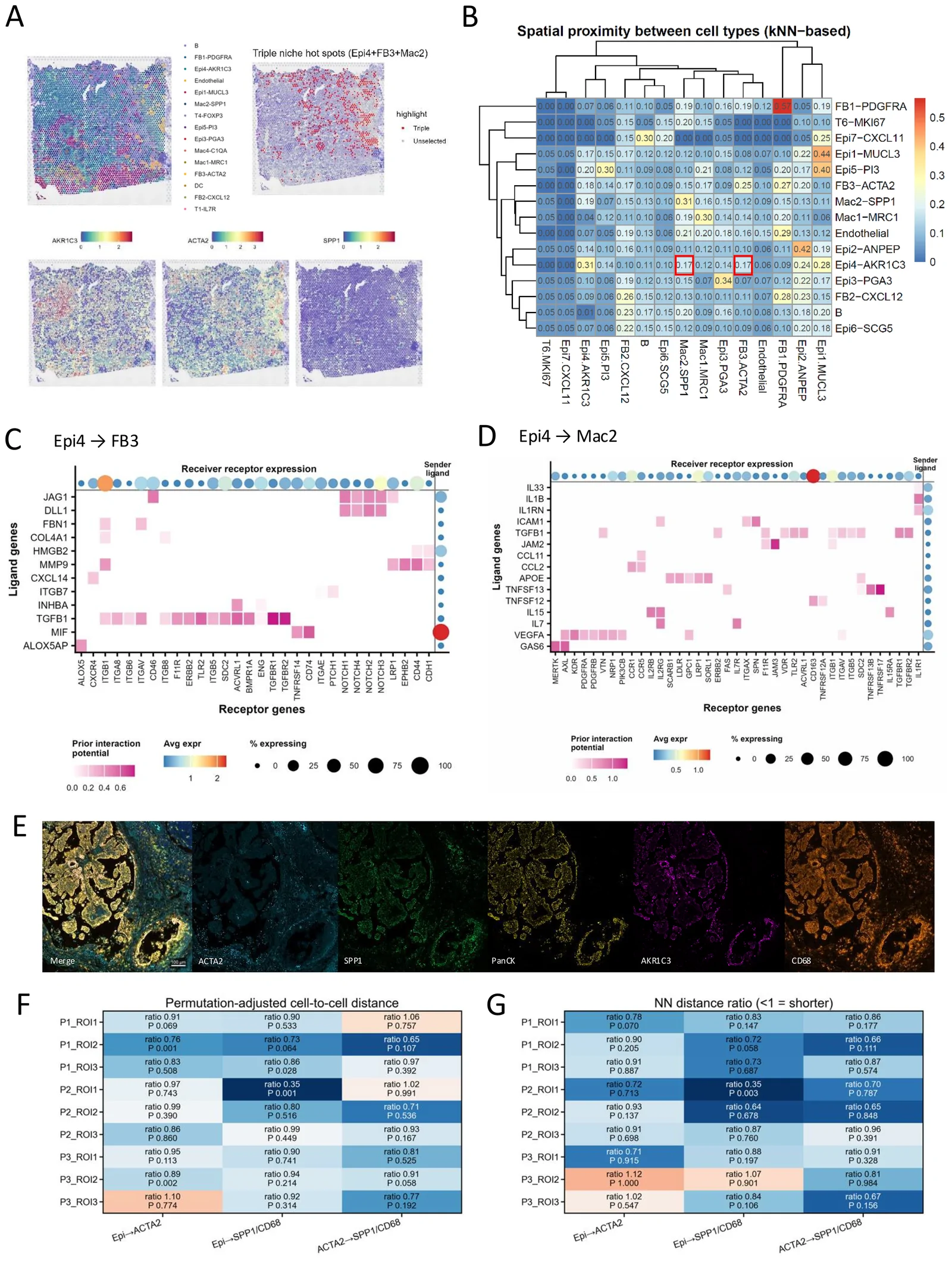
Descriptive spatial association and ligand-target prioritization for Epi4_AKR1C3, FB3_ACTA2, and Mac2_SPP1. **(A)** RCTD-estimated spatial abundances on a representative GSE251950 tumor section, with regions above the within-section 50th percentile for all three states and the distributions of AKR1C3, ACTA2, and SPP1 shown alongside. **(B)** k-nearest-neighbor (k = 10) proximity matrix across Visium sections. **(C)** NicheNet regulatory-potential interactions from Epi4_AKR1C3 to FB3_ACTA2. **(D)** NicheNet regulatory-potential interactions from Epi4_AKR1C3 to Mac2_SPP1. **(E)** Multiplex immunohistochemistry of GC tissue stained for Pan-CK, AKR1C3, ACTA2, SPP1, and CD68, with merged and single-channel views. Scale bar, 100 μm. **(F)** Permutation-adjusted cell-to-cell distance ratios among Pan-CK^+^/AKR1C3^+^ epithelial cells, ACTA2^+^ stromal cells, and SPP1^+^/CD68^+^ macrophages. **(G)** Nearest-neighbor (NN) distance ratios for the same cell pairs. Ratios < 1 indicate shorter-than-random spatial proximity; permutation-derived *P*-values are shown in each cell.

We next examined potential ligand-target pairs for the three states using NicheNet. In the Epi4_AKR1C3-to-FB3_ACTA2 analysis, TGFβ1-TGFβR1/TGFβR2 and JAG1/DLL1-NOTCH were among the prominent relationships (**Figure 5C**). ALOX5AP/ALOX5 was considered shared arachidonic-acid-related activity rather than a conventional ligand-receptor pair. For the Epi4_AKR1C3-to-Mac2_SPP1 comparison, GAS6-AXL/MERTK and VEGFA-KDR/NRP1 ranked highly, with CCL2-CCR1 and JAM2-JAM3 also present (**Figure 5D**). Because NicheNet predictions are based on expression data and do not show whether the corresponding cells are physically close in tissue, we used multiplex immunohistochemistry to examine marker localization at cellular resolution. In the representative GC field shown in **Figures 5E**, Pan-CK^+^/AKR1C3^+^ epithelial nests were located next to ACTA2^+^ myofibroblast cells and SPP1^+^/CD68^+^ macrophages along the epithelial–stromal interface. Across three independent patients and nine ROIs, quantitative distance analyses showed predominantly shorter-than-random distance ratios among the three marker-defined populations, although statistical support varied across ROIs and not all individual comparisons reached significance. This variability may reflect the pronounced spatial heterogeneity of the gastric TME. Overall, the results support a spatially heterogeneous epithelial–stromal–myeloid association at the proof-of-concept level (**Figures 5F, G**). These findings show that the three marker-defined populations can occur in close proximity within tumor environments.

## 4 Discussion

By combining single-cell and spatial transcriptomics with bulk-tissue survival analysis and multiplex immunohistochemistry, we examined how a particular gastric epithelial state relates to stromal and myeloid populations. Three states emerged as the main foci: Epi4_AKR1C3, FB3_ACTA2, and Mac2_SPP1. Epi4_AKR1C3 was nominally enriched in tumors and had an intermediate inferCNV burden, a distinct metabolic transcriptional profile, and an exploratory association with shorter overall survival. FB3_ACTA2 was also nominally enriched in tumors and displayed a myofibroblast-like program associated with stromal remodeling. Mac2_SPP1, in contrast, was not more abundant in tumor tissue but presented phagocytic and remodeling-related features. Spatial mapping placed the three states in overlapping tumor regions, while multiplex immunohistochemistry showed Pan-CK^+^/AKR1C3^+^ epithelial nests next to ACTA2^+^ stromal cells and SPP1^+^/CD68^+^ macrophages at the epithelial–stromal interface. These findings nominate a candidate epithelial–stromal–myeloid spatial configuration for further mechanistic testing.

Epi4_AKR1C3 is central to this candidate spatial association. AKR1C3 participates in prostaglandin and steroid metabolism and has previously been implicated in GC invasion and metastasis (14, 15). In the present analysis, the state was defined by more than AKR1C3 expression alone. It was nominally enriched in tumors and showed transcriptional enrichment of nicotinamide- and arachidonic-acid-related processes. These features raise the possibility that AKR1C3-positive epithelial cells occupy a metabolically distinct state that may influence the surrounding tissue through lipid-associated or redox-related processes. Arachidonic-acid-derived mediators are of particular interest because they can modify inflammatory polarization and immune regulation in tumors (14). Nevertheless, neither lipid flux nor metabolite secretion was measured. The ALOX5/ALOX5AP-related signal should therefore be regarded as shared pathway activity rather than a conventional ligand–receptor relationship.

FB3_ACTA2 represents the stromal component of the candidate spatial association. Fibroblast states can affect matrix architecture, immune exclusion, paracrine communication, and treatment response in GC (9–12). FB3_ACTA2 was the only fibroblast state nominally enriched in tumor tissue and was associated with vasculature development, cell adhesion, and developmental growth programs. This profile is consistent with a myofibroblast-like state involved in active stromal remodeling. NicheNet also ranked TGFβ1–TGFβR1/TGFβR2 and JAG1/DLL1–NOTCH among the candidate relationships between Epi4_AKR1C3 and FB3_ACTA2. The TGFβ1 result agrees with the established role of TGFβ-responsive myofibroblasts in ECM deposition and immunoregulatory stroma (10–12).

The transcriptional states identified in this study should be interpreted within the context of upstream regulatory mechanisms described in GC. AKR1C3, ACTA2, and SPP1 are therefore better regarded as representative markers rather than established causal drivers of the corresponding cell states. Previous ATAC-seq, enhancer-profiling, and massively parallel reporter assay (MPRA) studies have implicated RUNX/AP-1-associated regulatory activity, mesenchymal enhancer landscapes, and trans-acting transcription factors in shaping GC cell states (25–27). Consistent with these observations, our additional analysis showed limited direct overlap between the top state markers and previously reported regulatory genes, and also revealed state-biased expression of several upstream regulators (**Supplementary Figures S3C, D**). These findings support the view that the marker genes identified here may represent downstream manifestations of broader transcriptional and cis-regulatory programs.

Mac2_SPP1 exhibited a different pattern. Its relative abundance did not increase in tumor samples, but its bulk-tissue signature showed a nominal association with shorter overall survival in TCGA-STAD. The state was characterized by receptor-mediated endocytosis, phagocytosis, membrane invagination, and myeloid-cell activation. These features resemble previously described SPP1-positive tumor-associated macrophages involved in matrix remodeling and immune-restrictive niches (8, 13). The difference between abundance and survival association may be biologically informative. This contrast raises the possibility that the relevance of Mac2_SPP1 may be related to its functional program rather than simply to its abundance.

Spatial analysis brought the three independently identified states into a common tissue setting. RCTD detected tumor regions in which the estimated proportions of Epi4_AKR1C3, FB3_ACTA2, and Mac2_SPP1 were simultaneously high. The pairwise KNN results were more restrained. Visium spots also contain transcripts from multiple cells, so regional overlap cannot establish direct cell–cell adjacency. The mIHC image partly addresses this resolution problem by showing Pan-CK^+^/AKR1C3^+^ epithelial structures next to ACTA2^+^ myofibroblast-like stroma and SPP1^+^/CD68^+^ macrophages in a representative field. Taken together, these results support local spatial association, but they do not demonstrate a discrete or obligatory tri-cellular niche.

The adaptive immune findings provide additional context. B-cell composition was broadly similar between normal and tumor samples, whereas tumors showed modest enrichment of both T1_IL7R memory-like cells and T4_FOXP3 regulatory cells. The coexistence of memory- or activation-associated and regulatory programs argues against viewing the GC immune environment as uniformly activated or uniformly suppressed. None of the B- or T-cell signatures were nominally associated with survival in TCGA-STAD. This result directed the present analysis toward the epithelial–stromal–myeloid axis, but it does not exclude adaptive immune cells from the proposed model. These cells may still mediate some of the biological consequences of the candidate spatial association.

Several additional limitations should be considered and warrant further investigation. The survival associations of Epi4_AKR1C3, FB3_ACTA2, and Mac2_SPP1 were nominal. In multivariable Cox analyses adjusted for age, sex, tumor purity, and AJCC stage, only FB3_ACTA2 retained nominal statistical significance, whereas Epi4_AKR1C3 and Mac2_SPP1 did not. None of the three associations remained statistically significant after multiple-testing correction, indicating that these survival signals should be interpreted as exploratory. The available Visium dataset lacked matched spatially profiled normal gastric tissue, limiting our ability to determine whether the observed spatial configuration is specific to the tumor microenvironment. Multiplex immunohistochemistry was performed in only three patients, with three nested ROIs per case, and therefore provides only proof-of-concept cellular-resolution evidence of local proximity. Larger independent cohorts analyzed by quantitative multiplex imaging or higher-resolution spatial methods could determine whether AKR1C3-positive epithelial cells are consistently located near ACTA2-positive fibroblasts and SPP1-positive macrophages. Epithelial–fibroblast and epithelial–macrophage co-culture models could then test whether altering AKR1C3 changes fibroblast activation or macrophage polarization. Perturbation of TGFβ1–TGFβR1/2 and GAS6–AXL/MERTK (28, 29) would help to assess the predicted signaling relationships. Lipidomic analysis would also be valuable for determining whether the arachidonic-acid-related transcriptional program of Epi4_AKR1C3 corresponds to altered prostaglandin or other lipid-mediator production.

In summary, Epi4_AKR1C3, FB3_ACTA2, and Mac2_SPP1 represent transcriptionally distinct epithelial, stromal, and myeloid states that show regional co-enrichment in selected regions of the GC microenvironment. Their metabolic, stromal-remodeling, and phagocytic programs, together with exploratory survival associations and cellular-resolution histological adjacency, nominate an AKR1C3-associated epithelial–stromal–myeloid spatial configuration as a testable hypothesis for further mechanistic investigation.

## Data Availability

The original contributions presented in the study are included in the article/**Supplementary Material**. Further inquiries can be directed to the corresponding authors.

## Supplementary material

Supplementary Figure 1 B- and T-cell subpopulations in GC. **(A, B)** UMAPs of 21,408 B-cells and 42,555 T-cells. **(C, D)** Violin plots of marker genes. **(E, F)** Stacked bar charts of aggregate B- and T-cell subpopulation composition in N versus T samples. **(G, H)** Per-sample subpopulation comparisons using two-sided Wilcoxon rank-sum tests; nominal *P*-values are shown. T1_IL7R (*P* = 0.034) and T4_FOXP3 (*P* = 0.012) showed nominal tumor enrichment. **(I, J)** Mean expression heatmaps. **(K, L)** GO biological process terms passing BH-adjusted *P* < 0.05; dot color represents −log10 of the raw hypergeometric *P*-value.

Supplementary Figure 2 Multiple-testing correction of cell-state compositional comparisons. (A) Sample-level proportions of major cell lineages and epithelial, fibroblast, macrophage, B-, and T-cell states in normal (N) and tumor (T) samples. Differences were assessed using two-sided Wilcoxon rank-sum tests, followed by Benjamini–Hochberg correction for multiple comparisons; FDR-adjusted *P*-values are shown.

Supplementary Figure 3 Spatial and regulatory analyses of epithelial and candidate cell states. **(A)** Spatial randomization test for the Epi4_AKR1C3–FB3_ACTA2–Mac2_SPP1 tri-cellular KNN spatial-association score; the observed score exceeded the randomized null distribution (empirical *P* = 0.001). **(B)** Comparison of tri-cellular KNN spatial-association scores across alternative epithelial–fibroblast–macrophage combinations. **(C)** Overlap between the top-100 marker genes of Epi4_AKR1C3, FB3_ACTA2, and Mac2_SPP1 and previously reported GC regulatory genes. **(D)** Expression of literature-derived transcriptional regulators across the three states. Dot size indicates the percentage of expressing cells, and color indicates scaled average expression (z-score).
