# Supplementary material for: Spatial association of an AKR1C3-positive epithelial state with ACTA2-positive stromal cells and SPP1-positive macrophages in gastric cancer
Source: Front Oncol. 2026 Oct 28;16:1965632. doi: 10.3389/fonc.2026.1965632 (PMC13751489; doi:10.3389/fonc.2026.1965632)
Supplement: Supplementary file 1 [file Image1.pdf]

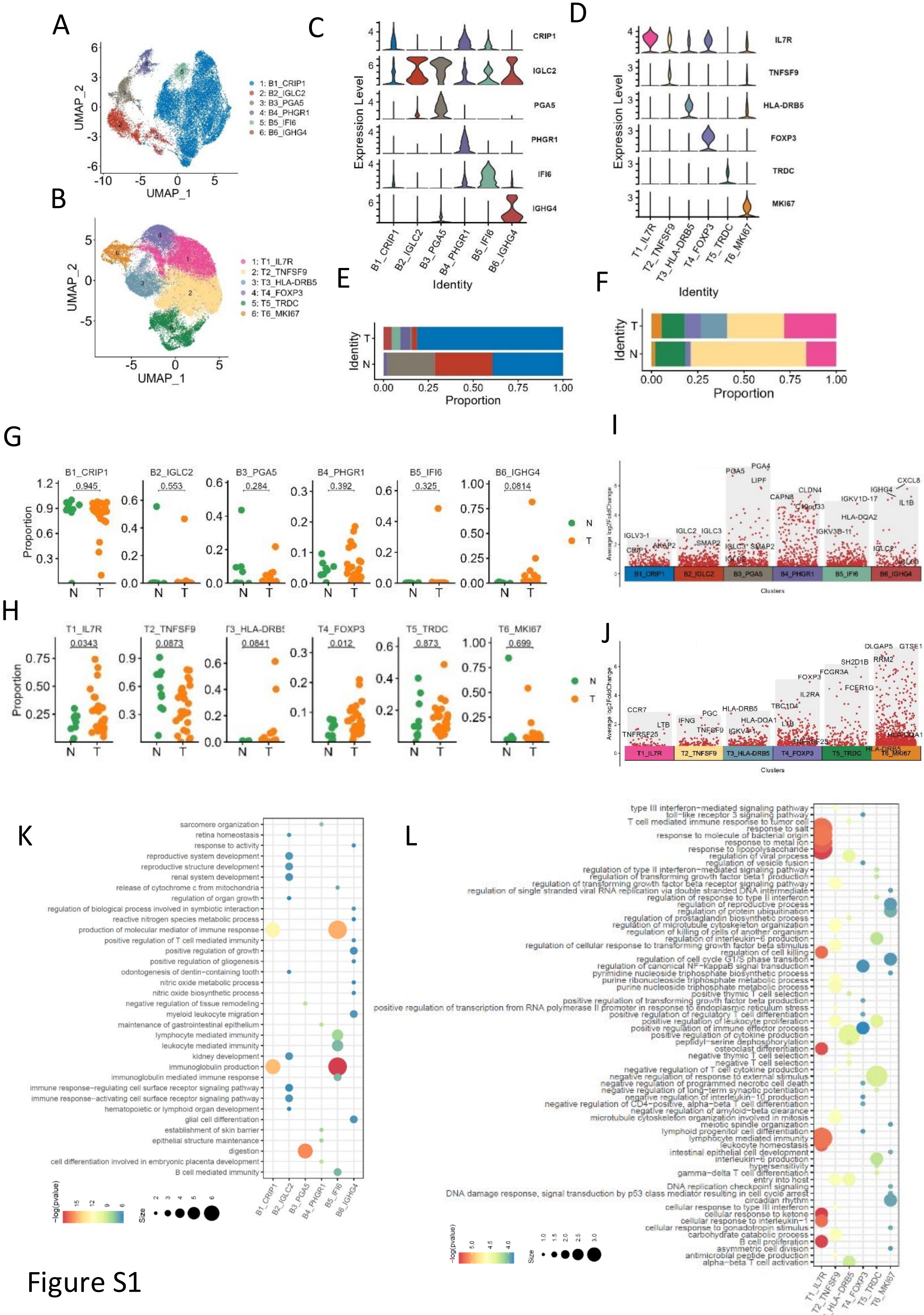

Figure S1
