# Supplementary material for: Spatial association of an AKR1C3-positive epithelial state with ACTA2-positive stromal cells and SPP1-positive macrophages in gastric cancer
Source: Front Oncol. 2026 Oct 28;16:1965632. doi: 10.3389/fonc.2026.1965632 (PMC13751489; doi:10.3389/fonc.2026.1965632)
Supplement: Supplementary file 2 [file Image2.pdf]

A

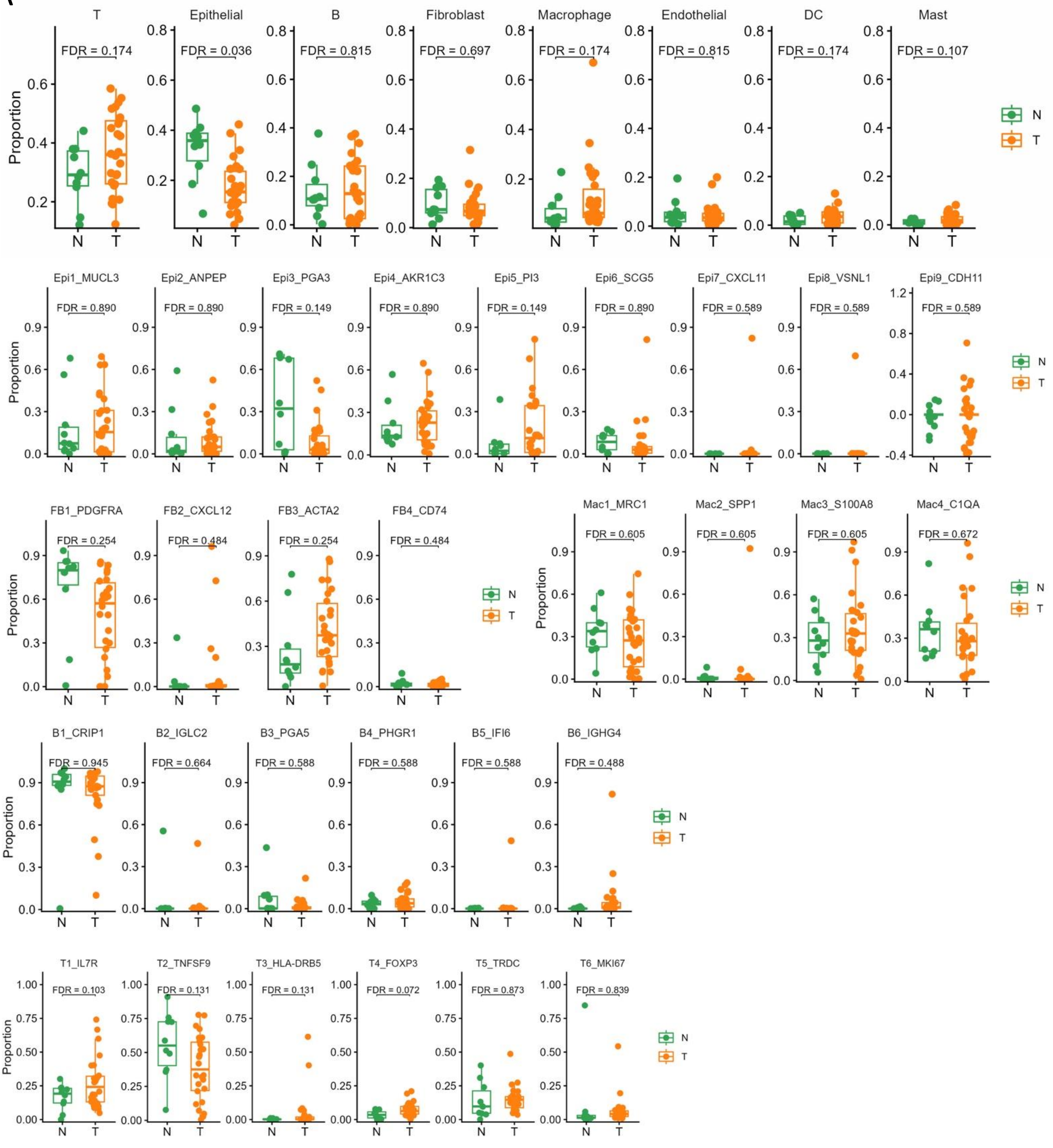
T
Epithelial
B
Fibroblast
Macrophage
Endothelial
DC
Mast
FDR = 0.174
FDR = 0.036
FDR = 0.815
FDR = 0.697
FDR = 0.174
FDR = 0.815
FDR = 0.174
FDR = 0.107
Proportion
N
T
Epi1_MUCL3
Epi2_ANPEP
Epi3_PGA3
Epi4_AKR1C3
Epi5_PI3
Epi6_SCG5
Epi7_CXCL11
Epi8_VSNL1
Epi9_CDH11
FDR = 0.890
FDR = 0.890
FDR = 0.149
FDR = 0.890
FDR = 0.149
FDR = 0.890
FDR = 0.589
FDR = 0.589
FDR = 0.589
FB1_PDGFRA
FB2_CXCL12
FB3_ACTA2
FB4_CD74
FDR = 0.254
FDR = 0.484
FDR = 0.254
FDR = 0.484
Mac1_MRC1
Mac2_SPP1
Mac3_S100A8
Mac4_C1QA
FDR = 0.605
FDR = 0.605
FDR = 0.605
FDR = 0.672
B1_CRIP1
B2_IGLC2
B3_PGA5
B4_PHGR1
B5_IFI6
B6_IGHG4
FDR = 0.945
FDR = 0.664
FDR = 0.588
FDR = 0.588
FDR = 0.588
FDR = 0.488
T1_IL7R
T2_TNFSF9
T3_HLA-DRB5
T4_FOXP3
T5_TRDC
T6_MKI67
FDR = 0.103
FDR = 0.131
FDR = 0.131
FDR = 0.072
FDR = 0.873
FDR = 0.839

Figure S2
