# Supplementary material for: Spatial association of an AKR1C3-positive epithelial state with ACTA2-positive stromal cells and SPP1-positive macrophages in gastric cancer
Source: Front Oncol. 2026 Oct 28;16:1965632. doi: 10.3389/fonc.2026.1965632 (PMC13751489; doi:10.3389/fonc.2026.1965632)
Supplement: Supplementary file 3 [file Image3.pdf]

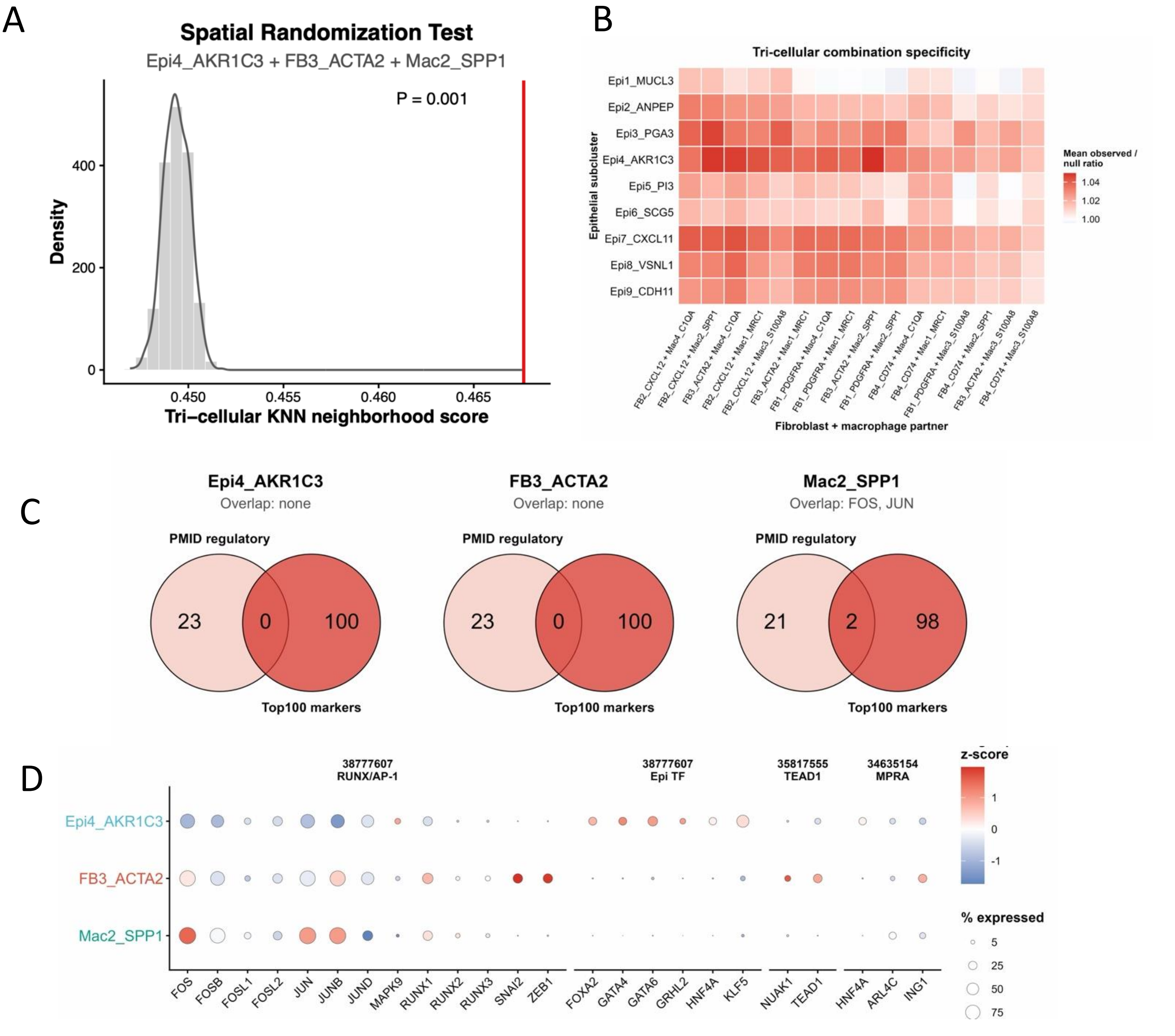
A
Spatial Randomization Test
Epi4_AKR1C3 + FB3_ACTA2 + Mac2_SPP1
P = 0.001
Density
Tri−cellular KNN neighborhood score
B
Tri-cellular combination specificity
Epithelial subcluster
Epi1_MUCL3
Epi2_ANPEP
Epi3_PGA3
Epi4_AKR1C3
Epi5_PI3
Epi6_SCG5
Epi7_CXCL11
Epi8_VSNL1
Epi9_CDH11
Mean observed / null ratio
Fibroblast + macrophage partner
C
Epi4_AKR1C3
Overlap: none
FB3_ACTA2
Overlap: none
Mac2_SPP1
Overlap: FOS, JUN
PMID regulatory
Top100 markers
D
38777607
RUNX/AP-1
38777607
Epi TF
35817555
TEAD1
34635154
MPRA
z-score
% expressed
Epi4_AKR1C3
FB3_ACTA2
Mac2_SPP1
FOS
FOSB
FOSL1
FOSL2
JUN
JUNB
JUND
MAPK9
RUNX1
RUNX2
RUNX3
SNAI2
ZEB1
FOXA2
GATA4
GATA6
GRHL2
HNF4A
KLF5
NUAK1
TEAD1
HNF4A
ARL4C
ING1

Figure S3
